# The Use of a Human Acellular Dermal Matrix (hADM) Transplant in Stage 4 Osteochondrosis Dissecans: A Retrospective Proof of Concept

**DOI:** 10.3390/jcm15197344

**Published:** 2026-09-22

**Authors:** Jannik Fußhoeller, Amelie Schild, Franziska Keßler, Jonas Neijhoft, Maren Janko, Johannes Frank, Ingo Marzi, René D. Verboket

**Affiliations:** Department of Trauma Surgery and Orthopaedics, University Hospital Frankfurt, Goethe University Frankfurt, 60590 Frankfurt, Germany; jannik.fusshoeller@unimedizin-ffm.de (J.F.); amelie.schild@unimedizin-ffm.de (A.S.); franziska.kessler@unimedizin-ffm.de (F.K.); jonas.neijhoft@unimedizin-ffm.de (J.N.); maren.janko@unimedizin-ffm.de (M.J.); johannes.frank@unimedizin-ffm.de (J.F.); ingo.marzi@unimedizin-ffm.de (I.M.)

**Keywords:** osteochondral lesion, talus, human acellular dermal matrix, hADM, Epiflex^®^, osteochondral reconstruction, FADI, cartilage repair, osteotomy

## Abstract

**Background**: Osteochondral lesions (OD) of the talus represent a significant clinical challenge, particularly in advanced-stage cases requiring osteotomy access and cancellous bone grafting. Human acellular dermal matrix (hADM) has demonstrated promising biological properties—including biocompatibility, preserved extracellular matrix architecture, and neovascularization capacity—in reconstructive bone and soft tissue surgery. To our knowledge, its use as a defect-sealing membrane in osteochondral surgery of the talus has not previously been reported clinically. **Methods**: This retrospective case series evaluated six patients with stage 4 talar OD treated surgically using hADM as a defect-closing membrane in combination with malleolar osteotomy and autologous cancellous bone grafting. Clinical outcomes were assessed using the Foot and Ankle Disability Index (FADI) and FADI Sport score at a mean follow-up of 13 months (range 10–14 months). Predefined safety endpoints comprised local inflammatory reaction, material-related adverse events, wound-healing disturbance, union of the malleolar osteotomy, iliac crest donor-site morbidity and revision surgery. **Results**: Functional improvement was observed in all six patients. The mean FADI score rose from 31.2 (±7.8) to 96.5 (±14.1) points and the mean FADI Sport score from 4.0 (±5.2) to 27.8 (±9.2) points, corresponding to absolute gains of 65.3 and 23.8 points, respectively. Five of six patients (83.3%) reported complete resolution of symptoms. No revision of the cartilage reconstruction was required, and none of the predefined adverse events occurred. **Conclusions**: In this uncontrolled six-patient series, sealing the reconstructed defect with hADM was technically feasible and none of the predefined adverse events were observed within the reported follow-up. Because all patients additionally underwent debridement, malleolar osteotomy and autologous cancellous bone grafting, the functional gains cannot be attributed to the membrane itself. These preliminary observations are hypothesis-generating and support further prospective, controlled investigation of hADM as a defect-sealing material in osteochondral surgery.

## 1. Introduction

### 1.1. Background

Osteochondrosis dissecans (OD) is a disease of the subchondral bone that classically affects adolescents and young adults, but is also encountered in middle-aged patients, in whom degenerative and post-traumatic osteochondral lesions of the talus predominate [1]. Progressive cartilage destruction may lead to the detachment of an osteochondral fragment [2]. Consequently, disruption of the joint architecture can occur, potentially resulting in the development of osteoarthritis [3].

The guidelines published in 2010 by the American Academy of Orthopedic Surgeons indicate that there is limited scientific evidence on the treatment of OD. Several considerations should be made for choosing the correct treatment options. Important factors in this consideration should include skeletal maturity and the stability of the lesion [4]. Especially in pediatric patients and in patients with small cartilage defects, conservative therapy seems to be a suitable option. This normally includes an immobilization of the affected joint and a reduction in physical activity [5]. In patients with unfavorable lesions or unsuccessful conservative therapy, surgical intervention should be considered [6].

### 1.2. Treatment Options

There is a wide range of different treatment options for osteochondrosis dissecans depending on the stage of the disease and the size of the lesion [7,8,9,10,11]. In smaller and more stable lesions and in pediatric patients, antegrade and retrograde drilling are common treatment options [12]. This leads to the activation of a natural remodeling process and induces local revascularization. The area of the created drill tunnel can be filled with autologous cancellous bone, which leads to primary static stabilization and supports the remodeling process [6,7,8,12,13]. In case of unstable lesions other treatment options should be considered [13]. Treatment principles involve the restoration of the joint surface, the fixation of the bone defects, and the improvement of blood flow to the affected area [14]. The choice of surgical procedure is mainly determined by the size of the defect. In the case of large defects, the debridement often creates an extensive bony defect zone, which must be filled with autologous cancellous bone [13]. If the dissecate is completely detached, therapy should always be carried out surgically with the aim of recovering the free joint body and treating the resulting defect [13]. Sole excision of the free joint body led to poor results, as well as an increased risk of osteoarthritis [15,16]. Refixation can be carried out if the dissecate is not too deformed. The choice of further surgical measures depends on the size and depth of the bone defect, whereby the bone quality of the affected area has a significant influence on the choice of treatment. Cartilage reconstruction can be carried out using various procedures. Because the ankle differs from the knee in cartilage thickness, joint congruency and loading pattern, the evidence derived from knee OD is only partially transferable to the talus; talus-specific data are therefore cited preferentially throughout this manuscript wherever such data exist [9,10,11,17,18].

The osteochondral autologous transplantation method (OATS) suggests taking a cartilage bone cylinder from a less stressed part of the joint and using it for transplantation [11,13,17]. Matrix-associated chondrocyte transplantation (MACT) is suitable for defect sizes of 2–4 cm^2^ [13,18]. MACT is a two-step surgical procedure in which autologous chondrocytes are harvested, expanded in vitro, and re-implanted within a biocompatible scaffold to repair articular cartilage defects [9]. In fact, there are already second-generation treatment options that can be performed in a single step. As an extension of MACT, techniques such as Autocart and minced cartilage transplantation have emerged to enhance cartilage repair by utilizing autologous cartilage fragments and biologics [10,19] (Table 1).

### 1.3. Closure of Defect Zone

All the procedures described have one thing in common. After an autotransplantation of chondrocytes or re-insertion of the dissecate, it is necessary to close the defect.

For the defect, the so-called Chondro-Gide^®^ membrane and a special thrombin solution [10,20] are used. The Chondro-Gide is a porcine transplant which only contains Collagen Type I and III [20].

Looking at osteochondral defects from a tissue engineering perspective, there are several necessities for successful regeneration and cell settlement in a collagen matrix [21]. In the literature, the human acellular dermal matrix (hADM) shows promising results in this context [22]: while it was originally used exclusively to cover skin defects, the application of human collagen matrices was investigated in 2024 for different possible use scenarios in clinical practice [22,23].

Focusing on modern and innovative treatment options, hADM also appears to be a suitable therapy candidate for advanced lesions addressing the cellular microenvironment and remodeling processes. Therefore, after receiving informed consent from the patients, we used hADM to cover defects in stage 4 OD situations in the talus that had to be treated by osteotomy, drilling out the defect, and filling the defect with cancellous bone. The filled defect was covered with hADM and fixed with fibrin glue. The results were reviewed retrospectively. The cases shown here are intended solely to demonstrate the technical feasibility of the application and to document the absence of specified adverse events. No comparative study was conducted, the functional scores are reported descriptively, and no claim of efficacy is made.

## 2. Materials and Methods

### 2.1. Study Description

Patients with ICRS stage 4 OD of the talus who underwent defect closure using human acellular dermal matrix (hADM; Epiflex^®^, DIZG, Berlin, Germany) between January 2020 and December 2024 were retrospectively analyzed. Inclusion criteria were a stage 4 osteochondral lesion of the talus according to the ICRS osteochondritis dissecans classification (ICRS OCD IV, i.e., an empty defect or a dislocated fragment), a lesion requiring malleolar osteotomy for adequate access, age ≥ 18 years, and no contraindication to open surgery. Exclusion criteria were concomitant ligamentous instability, active infection, inflammatory arthropathy, pregnancy, age < 18 years, previous surgery of the affected ankle including any previous cartilage repair procedure, severe hepatic or renal insufficiency, psychiatric disorders, alcohol or drug dependence, and refusal to receive a human allogeneic tissue product. Because hADM is derived from human donor skin, every eligible patient was informed of its allogeneic origin during the preoperative consultation; patients who declined implantation of human donor tissue were treated with a xenogeneic membrane instead and were therefore not included in this series. The series comprises all patients treated with hADM during the study period, that is, every eligible patient who consented to the implantation of a human allogeneic tissue product; the membrane was not selected by the surgeon on clinical grounds. Patients who declined were treated with one of the standard procedures. Their number was not recorded, so the proportion of eligible patients who received hADM cannot be stated, and the cohort is defined by patient consent rather than by a documented screening process.

### 2.2. Diagnostics

All patients answered the standardized Foot and Ankle Disability Index (FADI) questionnaire, which assesses and evaluates functional limitations related to foot and ankle disorders. It includes daily living activities and symptoms across multiple domains, including pain, stability, walking ability, and general functional performance. The FADI consists of 26 items: 22 items address functional activities and 4 items assess pain. Each item is scored on a 5-point Likert scale ranging from 0 (“unable to do” or “severe pain”) to 4 (“no difficulty” or “no pain”), whereas higher scores indicate better function and less disability. The highest possible score is 104. The FADI score has demonstrated strong reliability, validity, and responsiveness for clinical and research use in individuals with various foot and ankle pathologies [24,25,26,27].

As seen in Table 2, subjects were given the following instructions: “Please answer every question with one response that most closely describes your condition within the past week. If the activity in question is limited by something other than your foot or ankle, mark N/A.” Subjects rate the activity as no difficulty at all (4 points), slight difficulty (3 points), moderate difficulty (2 points), extreme difficulty (1 point), unable to do (0 points), or N/A (not applicable). For pain related to the foot and ankle, subjects select no pain (4 points), mild (3 points), moderate (2 points), severe (1 point), or unbearable (0 points). The Foot and Ankle Disability Index is reported as having a raw point score with a maximum of 104 points, and the Foot and Ankle Disability Index Sport as having a raw point score with a maximum of 32 points. All values reported in this manuscript are raw point scores, not percentages (Table 2).

The FADI Sport score is an adjunct subscale developed to capture higher-level functional demands relevant to athletic and physically active populations. It consists of 8 items that assess activities such as running, jumping, lateral movements, and quick direction changes. Items are scored using the same 0–4 Likert scale as the FADI with a possible maximum score of 32. Higher scores reflect better sport-related functional abilities. The FADI Sport demonstrates good reliability and sensitivity to changes in athletic performance and is frequently used to evaluate recovery in individuals returning to sport following foot or ankle injury.

In addition, we documented the rehabilitation process and included observations made during postoperative care, such as wound healing and the need for further surgery. The FADI and FADI Sport questionnaires were completed by the patients themselves as part of routine clinical care, preoperatively and postoperatively at the routine follow-up examination, to which patients were invited and reminded. No score was reconstructed from recall at a later date; the scores were thus documented prospectively within routine care and analyzed retrospectively. All clinical follow-up was carried out at the treating institution: patients were reviewed every two weeks during the first four postoperative weeks, every four weeks until the sixth postoperative month, and every two months thereafter. No assessment was performed externally and no patient was lost to follow-up. MRI and X-ray controls were also performed in terms of standard care (Table 3). Cartilage repair tissue was morphologically assessed in five of the six patients using magnetic resonance imaging (MRI) and evaluated according to the MOCART 2.0 score [28]. Imaging was performed on 3 Tesla scanners (MAGNETOM Prisma Fit and MAGNETOM Vida, Siemens Healthineers, Erlangen, Germany). The protocol comprised sagittal and coronal proton-density-weighted turbo spin-echo sequences with fat saturation (TR 2100 ms, TE 25 ms, field of view 100 mm, acquisition matrix 320, in-plane resolution 0.31 × 0.31 mm) and a sagittal T1-weighted turbo spin-echo sequence without fat saturation (TR 788 ms, TE 9.7 ms, field of view 150 mm, acquisition matrix 384, in-plane resolution 0.39 × 0.39 mm). All sequences were acquired with a slice thickness of 2.0 mm. MRI was obtained at a mean of 12.6 months postoperatively (range 11–14 months); the individual imaging intervals are given in Table 3. One patient declined a further MRI examination because of claustrophobia and was therefore not included in the MOCART analysis; no imaging was withheld or excluded on the basis of its findings. This score comprises seven variables—volume fill of the (osteo)chondral defect, integration into the adjacent cartilage and bone, surface of the repair tissue, signal intensity of the repair tissue, bony defect or bony overgrowth, edema-like marrow signal, and subchondral cysts—which were summed to yield a total score ranging from 0 to 100 points, with higher scores indicating a more favorable morphological outcome. Scoring was performed in accordance with the published grading criteria by two reviewers who assessed the examinations independently and were blinded both to the clinical outcome and to each other’s ratings. The two readers agreed exactly on 31 of the 35 item ratings (88.6%); the four discrepant items were resolved by consensus between the two readers, and the consensus values are reported. Agreement on the total score was good, with an intraclass correlation coefficient of 0.87 (two-way random effects, absolute agreement, single measures) and a mean absolute difference between readers of 5.0 points (range 0–10). Given that there were only five examinations, these figures are descriptive and should not be generalized.

### 2.3. hADM (Epiflex^®^)

The hADM Epiflex^®^ is human acellular dermis which is obtained from donated human skin and processed under strict control and following EudraLex guidelines for good manufacturing practice conditions [29]. The manufacturing process involves complete decellularization while preserving the ultrastructural architecture of the extracellular matrix. This effectively removes immunogenic components while maintaining the native collagen organization, resulting in very high biocompatibility and low immunogenicity [22,23].

The hADM contains human collagen type I, III, IV, fibronectin, laminin, vitronectin, hyaluronic acid and unlike the Chondro-Gide the collagen structure has been kept [23]. This ensures optimal support for cell growth, migration and tissue regulation [22,30]. As a central component of the extracellular matrix (ECM), the collagen matrix critically regulates biochemical signaling by providing specific binding sites for growth factors and cytokines, thereby playing a fundamental role in tissue engineering [22,31,32].

From a biophysical perspective, the hADM exhibits high diffusion permeability to oxygen and water [23], a property that is particularly beneficial within the defect zone, as it ensures adequate oxygen diffusion while still supporting chondrocyte adhesion to the matrix [22]. Following local cell migration and progressive remodeling of the defect site, the implanted membrane undergoes gradual resorption [22]. Importantly, human acellular dermis demonstrates favorable mechanical properties, including high tensile strength and structural stability, which are largely preserved under physiological loading conditions, as evidenced by experimental in vitro studies [33].

After implantation, hADM functions as a biologically active guiding structure. Preclinical studies have demonstrated rapid vascularization of the matrix and its colonization by host-derived cells, including fibroblasts and mesenchymal stromal cells [34,35]. These characteristics facilitate functional integration of the membrane into the surrounding tissue and generate a pro-regenerative microenvironment. Moreover, the collagen matrix provides specific binding sites for cell receptors such as integrins and discoidin domain receptors, which can support cell adhesion, migration and differentiation [22]. Collectively, these properties suggest that hADM holds considerable potential for broader application in the field of reconstructive cartilage surgery.

### 2.4. Procedure Description

The procedure for the treatment of OD of the talus was performed in supine position, ensuring optimal exposure of the ankle joint. The incision follows the medial aspect of the ankle to expose the medial malleolus. After standardized medial malleolar osteotomy, the defect was debrided to bleeding bone, filled with 4–6 cc autologous iliac cancellous graft, harvested from the ipsilateral iliac crest, and sealed with hADM. A graft measuring 15 × 15 mm with a thickness of at least 0.8 mm was used and rehydrated in sterile 0.9% sodium chloride solution in two consecutive baths of five minutes each. The membrane carries a macroscopically evident polarity, so that its orientation can be determined reliably on the operating table without additional marking: the porous surface shown in Figure 1, which corresponds to the former subcutaneous aspect, was placed towards the cancellous bone graft, and the smooth former epidermal surface towards the joint. The membrane was trimmed to the outline of the defect and inserted flush with the surrounding cartilage surface, without overlapping the native cartilage. It was fixed with 1 mL of fibrin sealant (TISSUCOL Duo S Immuno, Baxter, Unterschleißheim, Germany). The sealant was applied with a spray applicator. Before the reduction of the osteotomy, the ankle was moved through its range of motion intraoperatively to confirm that the membrane remained in position. Then the osteotomy was reduced and stabilized using two cannulated screws. Postoperatively, the ankle was immobilized in a splint for two weeks and then changed to a functional orthosis for a further four weeks. Range-of-motion exercises were begun cautiously after the first two weeks and were performed without loading. Sole-contact (touch-down) weight-bearing of the operated limb was maintained for six weeks; progressive loading was then started under physiotherapeutic supervision, once radiographic control had confirmed the consolidation of the medial malleolar osteotomy, and full weight-bearing was usually reached at eight weeks. Pharmacological thromboprophylaxis was continued until full weight-bearing had been achieved. Return to sporting activity was recommended from twelve weeks postoperatively.

### 2.5. Statistics

The collected data were analyzed with Statistical Package for the Social Sciences (SPSS) version 26.0 (IBM Corp., Armonk, NY, USA) for descriptive statistical analysis.

Frequencies are presented as absolute values and percentages, rounded to one decimal place. Descriptive statistics were performed to summarize and characterize the study data, providing a snapshot of central tendencies and data spread. Metric variables were expressed as arithmetic mean ± standard deviation (SD). Given a sample size of six patients, no inferential statistical testing was performed; accordingly, no *p*-values, confidence intervals or effect estimates are reported, and all comparisons between preoperative and postoperative scores are descriptive. Because measures of central tendency are of limited informative value at this sample size, individual patient values are additionally reported (Table 3) and score changes are given in absolute points rather than as percentages.

### 2.6. Ethics

This study was performed retrospectively at the University Hospital of the Goethe University Frankfurt with the institutional ethical approval in accordance with the Declaration of Helsinki (2026-2773). Epiflex^®^ is licensed as a medicinal product (Arzneimittel) under German law and was applied as part of routine clinical care; the study was not an investigational trial of the device. Written informed consent for the procedure was obtained from every patient, and each patient was separately informed about, and consented to, the implantation of a human allogeneic tissue product.

## 3. Results

A total of six patients were included in this retrospective analysis. The mean age at surgery was 45.8 ± 18.2 years (range 22–64 years), and four of the six patients were male (66.7%). The cohort therefore comprised both young adults and patients in the sixth and seventh decades of life. In five of the six cases (83.3%) the right ankle was affected. All lesions were located on the medial shoulder of the talus and were stage 4 OD requiring osteotomy access. None of the patients had undergone previous surgery of the affected ankle, and none had received a previous cartilage repair procedure (Table 4). The lesion was post-traumatic in four patients and idiopathic in two. The two idiopathic lesions occurred in the youngest patients of the cohort, aged 22 and 27 years, whereas all four patients older than 40 years had a post-traumatic lesion. Symptom duration, body mass index, smoking status and the presence of diabetes mellitus were not documented consistently in the clinical records and could not be reconstructed retrospectively; these variables are therefore not reported. The extent of the defect was determined preoperatively by MRI. In the patient cohort presented here, it amounted to 3.93 (+/−0.91) ccm. Defect volume was measured on the preoperative MRI by manual segmentation of the lesion on consecutive slices. Volume rather than area was chosen deliberately: these were deep, bony defects requiring cancellous grafting, in which the volume of the graft determines the extent of the reconstruction, whereas an area measurement does not capture the subchondral component and would understate the size of the defect actually treated. Lesion area and depth were not recorded separately and could not be reconstructed from the retrospective imaging reports. We acknowledge that this limits direct comparison with the talar literature, which conventionally reports on the lesion area. Individual volumes are given in Table 3.

The average preoperative Foot and Ankle Disability Index (FADI) score was 31.17 (±7.8), indicating substantial limitations in daily activities. The preoperative FADI Sports sub-score, which reflects sport-specific functionality, averaged 4.0 (±5.22), suggesting severely impaired physical performance levels.

### Outcome

Clinical and functional follow-up was performed at a mean of 13 months (range 10–14 months) postoperatively. All six patients improved in both scores. The mean postoperative FADI score was 96.5 (±14.12; median 103, range 68–104), corresponding to an absolute increase of 65.3 points, and the mean FADI Sport score was 27.83 (±9.24; median 31.5, range 9–32), corresponding to an absolute increase of 23.8 points. The comparatively wide standard deviation of the postoperative FADI score is attributable to a single patient (patient 4, 68 points); the remaining five patients scored between 98 and 104 points. Individual absolute changes ranged from 40 to 80 points for the FADI and from 6 to 32 points for the FADI Sport score (Table 5). Relative changes are not reported: the very low preoperative baselines—in particular a mean FADI Sport score of 4.0 of 32 attainable points—make percentage increases arithmetically unstable and clinically uninformative. To our knowledge, no minimal clinically important difference has been validated for the FADI or FADI Sport score in patients with osteochondral lesions of the talus, so the observed changes cannot be related to such a threshold (Table 4).

Five of the six patients (83.3%) reported complete freedom from complaints. The patient who still experienced symptoms after surgery (patient 4) had the largest preoperative defect (5.02 cm^3^), the lowest postoperative scores (FADI 68, FADI Sport 9) and the lowest MOCART 2.0 score (45 points) and was the only patient with persistent end-range limitation of motion. The mean duration of the surgical procedure was 73.8 min (±16.6).

No patient required a revision of the cartilage reconstruction. Elective removal of the osteotomy screws was performed in four of the six patients 12 to 13 months after the index operation; in one patient, removal has not yet been carried out and in one patient it was declined. Hardware removal after malleolar osteotomy is an anticipated second procedure rather than a treatment failure and is therefore reported separately from revision of the cartilage reconstruction. Radiographic union of the medial malleolar osteotomy was achieved in all six patients, and no donor-site complications at the iliac crest—persistent pain, hematoma, infection or sensory disturbance—were recorded. None of the predefined safety endpoints occurred: there was no local inflammatory reaction of the ankle joint, no adverse reaction attributable to the implanted allogeneic material, no wound-healing disturbance and no delayed union. The mean number of postoperative consultations including dressing changes was 5.8 (±5.3); this figure is reported descriptively and is not used as a surrogate measure of safety (Table 5).

The mean MOCART 2.0 Ankle score of the five patients with available MRI was 65 ± 9.4 points (median 65, range 50–75). Individual total scores are given in Table 3 and the complete distribution of all seven subcategories in Table 6. The defect was completely filled in one patient and showed minor underfilling (50–99%) in four; severe underfilling or chondro-osseous delamination was not observed. Integration into the adjacent cartilage and bone showed a fluid-like split of 2 mm or less in all five patients, confined to the cartilage in two and extending to the bone in three; complete integration was not achieved in any patient. The surface of the repair tissue was intact in three patients and irregular in two. The signal intensity of the repair tissue was mildly abnormal in all five patients and normal in none. Two patients showed neither a bony defect nor overgrowth, while three had a residual bony defect of less than 5 mm or an overgrowth of less than 50%. An edema-like marrow signal was present in every patient, minor in four and severe in one, and subchondral cysts smaller than 5 mm were present in four patients. The lowest score (50 points) was recorded in patient 4, who also had the largest preoperative defect and the lowest functional scores. A mean of 65 of the 100 attainable points indicates partial, not complete, structural restitution and should not be interpreted as evidence of cartilage regeneration (Table 6).

## 4. Discussion

This retrospective analysis evaluated the clinical outcomes of using a human acellular dermal matrix (hADM) to close the defect zone in the treatment of osteochondral lesions (OD) of the talus. The sample size was limited to six patients and no control group was available, so the analysis is descriptive throughout. A marked improvement of both the FADI and the FADI Sport score was observed at a mean of 13 months (range 10–14 months), with absolute gains of 65.3 and 23.8 points, respectively. Five of the six patients (83.3%) reported complete resolution of symptoms and none required revision of the cartilage reconstruction. Because every patient underwent debridement, malleolar osteotomy and autologous iliac crest cancellous grafting in addition to the hADM coverage, these gains reflect the combined effect of a multimodal procedure and cannot be attributed to the membrane: the series shows that the construct is feasible, not that the membrane works. The follow-up is far too short to permit any statement about durability.

The preoperative FADI score of 31.17 ± 7.8 indicates severe functional limitation, notably lower than most reported series for talar osteochondral lesions [36,37]. This discrepancy likely reflects our strict inclusion of advanced lesions (stage 4 OD requiring osteotomy access and defect size 3.93 ± 0.91 cm^3^), which typically present with greater subchondral collapse and cartilage destruction than arthroscopically managed defects in comparative studies [38,39]. The mean MOCART 2.0 Ankle score of 65 points is of the same order as the values reported after AMIC for talar osteochondral lesions and, like those series, reflects partial rather than restitutional repair [40,41]. Accordingly, these results must be interpreted within the context of a surgically challenging patient cohort, and the observed improvements may be particularly relevant for cases in which conventional arthroscopic techniques are insufficient. These observations indicate that hADM can be used as an adjunctive defect-sealing material within reconstructive techniques for severe OD, particularly in complex cases requiring osteotomy and cancellous bone grafting; whether it confers any benefit over established membranes remains an open question (Figure 2).

### 4.1. Possible Use of hADM (Epiflex^®^)

The hADM has been clinically established for years as an allogeneic dermal replacement matrix, particularly in reconstructive surgery for the treatment of complex skin and soft tissue defects and in breast reconstruction [42,43,44]. Compared to xenogeneic collagen matrices, human acellular dermal matrices have been reported to have lower complication rates and very good tolerability, which is attributed to their high structural similarity to the human extracellular matrix [42,44].

In recent years, the hADM has also gained increasing importance in reconstructive bone and soft tissue surgery—for instance, as an experimental replacement for the induced membrane in the classic Masquelet procedure [35]. The underlying mechanisms appear to be similar to those of the induced membrane: the immediately effective barrier function prevents fibrotic soft tissue ingrowth into the defect area, thereby reducing the risk of pseudarthrosis formation. In addition, preclinical studies have demonstrated pronounced vascularization of the implanted hADM, indicating biological activity of the membrane comparable to that of the induced Masquelet membrane [22,34,35].

Clinical experience with hADM to date is confined to soft tissue and breast reconstruction; in bone, the available evidence is preclinical. In a rat femoral critical-size defect model, a single-stage membrane technique using hADM achieved complete radiological healing in all treated animals and a biomechanical strength comparable to that of the induced Masquelet membrane, without relevant adverse reactions [35]. Histological analyses in the same model showed partial integration of the membrane into newly formed bone tissue and pronounced neovascularization within the matrix. These findings are preclinical and derived from a bone defect model; they cannot be transferred directly to loaded articular cartilage [33,35].

The neovascularization observed in experimental bone tissue healing with hADM may theoretically benefit the subchondral bone component of ODs, given that impaired vascularity is considered a key contributor to subchondral necrosis and delayed healing in osteochondral pathology. However, whether hADM simultaneously facilitates chondrocyte migration and cartilage regeneration remains speculative and requires direct histological validation in osteochondral tissue. In theory, as ODs often present with subchondral necrosis combined with cartilage fragmentation, hADM may offer an ideal interface for facilitating integration of bone grafts while simultaneously providing a supportive scaffold for chondrocyte migration and repair processes [6,22]. Beyond its structural role, the preserved extracellular matrix architecture of hADM—including collagen types I and IV, fibronectin, and laminin—may provide biological cues that actively promote cellular infiltration and tissue remodeling, rather than functioning solely as a passive barrier [22,31,32]. These properties may distinguish hADM from synthetic or xenogeneic alternatives and represent the rationale for its continued investigation in osteochondral surgery. It must be emphasized that no histological or other direct biological evidence of chondrocyte migration, cartilage formation or matrix vascularization was obtained in the patients reported here. The mechanisms outlined above are extrapolated from dermal, soft tissue and bone defect models and from in vitro work; in the context of loaded articular cartilage they remain hypotheses that require direct histological validation [22,30,35].

In this context, a comparison with the established AMIC technique is instructive. AMIC employs a collagen type I/III bilayer scaffold (Chondro-Gide^®^) to seal the defect following a microfracture, thereby creating a contained environment for mesenchymal stem cell differentiation and fibrocartilaginous repair tissue formation—a conceptually analogous mechanism to the barrier and scaffold functions proposed for hADM. In the landmark study by Valderrabano et al. (2013), 26 patients with advanced talar OD underwent an open AMIC procedure combined with autologous cancellous grafting, with clinical and radiological assessment performed at a minimum of 24 months postoperatively [40]. The AOFAS score improved from a mean of 60 points (range 17–79) preoperatively to 89 points (range 61–100) at follow-up, and the MOCART score averaged 62 points (range 20–95), demonstrating satisfactory mid-term functional and structural outcomes. Similarly, Usuelli et al. (2018) reported on 20 patients treated with an all-arthroscopic AMIC technique combined with autologous bone grafting for type III and IV talar lesions, demonstrating a significant improvement in AOFAS score from 57.1 ± 14.9 preoperatively to 86.6 ± 10.9 at 24 months, along with meaningful reductions in lesion area on both CT and MRI [45]. In a broader analysis, a systematic review pooling data from 778 patients treated with AMIC for talar OD reported significant improvements in VAS, AOFAS, and FFI scores, with a revision surgery rate of 7.8% and a failure rate of 6.2% [46].

Taken together, these AMIC data provide a meaningful benchmark for interpreting our hADM results. While direct comparison is limited by differences in scoring systems (FADI vs. AOFAS), defect severity, and surgical technique, the magnitude of functional improvement observed in our hADM cohort falls within the range reported after AMIC. This is contextual information, not comparative evidence of efficacy: the studies differ in sample size, outcome instrument (FADI versus AOFAS or FFI), defect measurement (volumetric versus area-based), lesion stage, surgical technique and follow-up duration, and an uncontrolled series of six patients followed for 10–14 months cannot support any claim of equivalence or superiority. Importantly, however, the collagen scaffold used in AMIC has an established evidence base with histological validation and longer follow-up data, which hADM currently lacks in the osteochondral context. Whether hADM offers biological advantages over xenogeneic collagen matrices in terms of vascularization, host integration, and chondrogenic support can only be determined through controlled comparative studies.

### 4.2. Limitations

Several limitations of this study must be taken into account. First, the retrospective study design represents a relatively low level of evidence and is susceptible to selection and reporting biases inherent to this methodology. Furthermore, due to the narrow indication for the procedure, and the need of special patient informed consent and preoperative briefing using a new technique the sample size was very small (*n* = 6), which limits statistical power and means that rare complications could not be reliably detected or excluded. Accordingly, the absence of adverse events in this series should be read as the absence of the specified events in six patients over 10–14 months, not as evidence of a favorable safety profile. A further consequence of the retrospective design is that symptom duration and patient-level comorbidity data—body mass index, smoking status and diabetes mellitus—were not documented consistently enough to be retrieved. The cohort therefore cannot be characterized in these respects, and potential confounding by these factors cannot be assessed; prospective studies should record them from the outset. A further constraint applies to cohort selection: the six patients are those who consented to receive a human allogeneic tissue product, and the number of eligible patients who declined and were treated by a standard procedure was not recorded, so the degree of selection cannot be quantified. The functional scores themselves were documented prospectively as part of routine care and are therefore not affected by recall bias, but they were obtained at a variable postoperative interval of 10 to 14 months rather than at a fixed protocol time point.

Our study also lacked a control group, making it impossible to draw direct conclusions about how hADM compares with other established techniques. A controlled comparison with AMIC would be particularly relevant. Weigelt et al., in a 2- to 8-year follow-up of 33 patients treated with AMIC for talar osteochondral lesions, reported no revision surgery for failed AMIC but a reoperation rate of 58%, predominantly for symptomatic hardware following medial malleolar osteotomy [47]. Our own cohort is consistent with that observation: four of the six patients underwent elective screw removal within 12 to 13 months, a proportion in the same range as the 58% reported by Weigelt et al. Any account of this approach that reports only the absence of revision surgery therefore understates the number of times these patients return to the operating room. Our follow-up of 10–14 months nevertheless remains too short to capture late failure of the cartilage reconstruction itself. Additionally, the use of different patient-reported outcome measures across studies (FADI in our cohort vs. AOFAS or FFI in most AMIC studies) substantially hampers direct numerical comparisons and underscores the need for standardized outcome reporting in future osteochondral research.

Furthermore, no standardized postoperative MRI at the same time point for all patients was performed in this cohort or available for all patients, nor was any histological analysis conducted. Objective imaging assessment—particularly quantitative MRI sequences such as T2 mapping or dGEMRIC—at 12 and 24 months could reveal structural differences in cartilage and subchondral bone quality and individual progress that functional outcome scores alone cannot capture. The absence of such data for different timepoints (e.g., 12 and 24 months) in our series represents a relevant gap that future investigations with hADM should address.

Finally, the study is based on short- to medium-term follow-up observations. It is therefore not possible to draw conclusions about long-term durability of clinical improvement or the potential influence of the procedure on the development of secondary osteoarthritis—a particularly important endpoint in this indication.

### 4.3. Prospects

The encouraging clinical outcomes observed in this retrospective analysis support further systematic investigation of hADM in the treatment of osteochondral lesions. Future research should prioritize prospective, randomized controlled trials comparing hADM with established cartilage reconstruction techniques, such as AMIC or OATS, to determine its relative efficacy and safety profile. Such studies should include larger, adequately powered patient cohorts, standardized surgical protocols, and clearly defined inclusion and exclusion criteria to improve the comparability, internal validity, and generalizability of results. The adoption of uniform outcome instruments—ideally including both region-specific scores such as the FADI and widely used global measures such as the AOFAS or PROMIS—would further facilitate cross-study comparisons with the existing AMIC literature.

In addition to patient-reported clinical outcome measures, future trials should incorporate advanced imaging modalities—including quantitative MRI techniques such as T2 mapping and MOCART scoring after 12 and 24 months—to objectively assess cartilage regeneration, subchondral bone remodeling, and integration of the implanted matrix over time, in line with established reporting standards in the AMIC literature. Longer follow-up periods of at least five to ten years will be essential to evaluate the durability of clinical improvement and to determine whether hADM-augmented reconstruction influences the natural history of post-traumatic osteoarthritis in the ankle.

Furthermore, translational and basic science studies investigating the biological mechanisms of hADM integration—including its vascularization kinetics, interaction with subchondral bone, and potential to support chondrocyte migration and differentiation—may help to better define its mechanistic role in osteochondral reconstruction and guide the future optimization of surgical technique and implant design.

### 4.4. Summary

In summary, the human acellular dermal matrix appears to be a promising biomaterial for closing defect zones in osteochondral surgery. Its favorable biological properties—including high biocompatibility, preservation of the native extracellular matrix architecture, and demonstrated capacity for vascularization—support its use as an interface material in complex osteochondral reconstructions requiring osteotomy access and cancellous bone grafting.

In the present series, the use of hADM as a defect-sealing membrane was technically feasible and was not associated with any of the predefined adverse events. Because the membrane was only one component of a multimodal procedure, the functional improvement observed cannot be attributed to hADM. The observed magnitude of functional improvement lies within the range of published outcomes of the established AMIC technique; given the differences in study design, lesion severity and outcome measures, this is contextual information rather than comparative evidence. No hADM-related adverse event was observed, which is consistent with the established biocompatibility profile of this matrix in other reconstructive applications. With six patients and a follow-up of 10–14 months, however, this cohort is far too small to detect uncommon complications or to establish a safety profile.

However, despite these encouraging preliminary findings, the current evidence base remains limited by the retrospective design, small cohort size and absence of comparative data. Further prospective and ideally randomized controlled studies—with standardized outcome reporting and sufficient follow-up—are needed to validate these results, compare hADM with established closure materials and cartilage repair techniques including AMIC, and define its long-term role in cartilage and osteochondral repair. Only through such rigorous investigation can the full clinical potential of this approach be established and its integration into evidence-based treatment algorithms be justified.

## Figures and Tables

**Figure 1:**
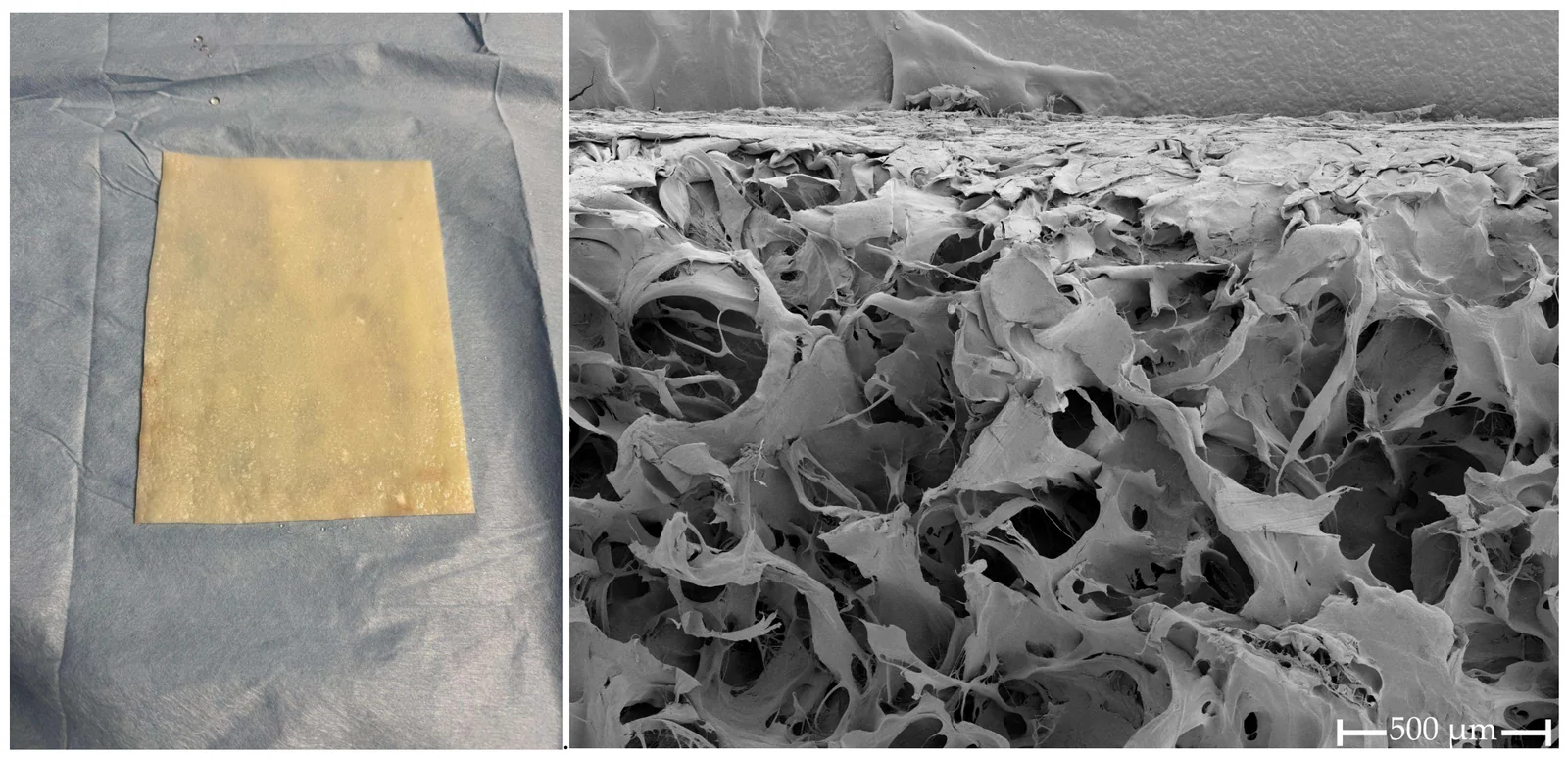
Representative image of the hADM graft. Prior to implantation, the matrix is hydrated to restore its native structural properties. Consistent with its dermal origin, the membrane exhibits a distinct polarity, with one smooth surface corresponding to the former epidermal side and the opposite, porous surface representing the subcutaneous aspect. In the hydrated state shown, the membrane combines tear resistance with flexibility, allowing precise adaptation and conformal shaping to the defect site and a scanning electron microscope image of the hADM membrane, clearly showing its multilayer structure and the membrane’s polarity (Picture top is a dermis-side view). Both images were created in the authors’ own laboratory; the manufacturer supplied neither the images nor any technical assistance.

**Figure 2:**
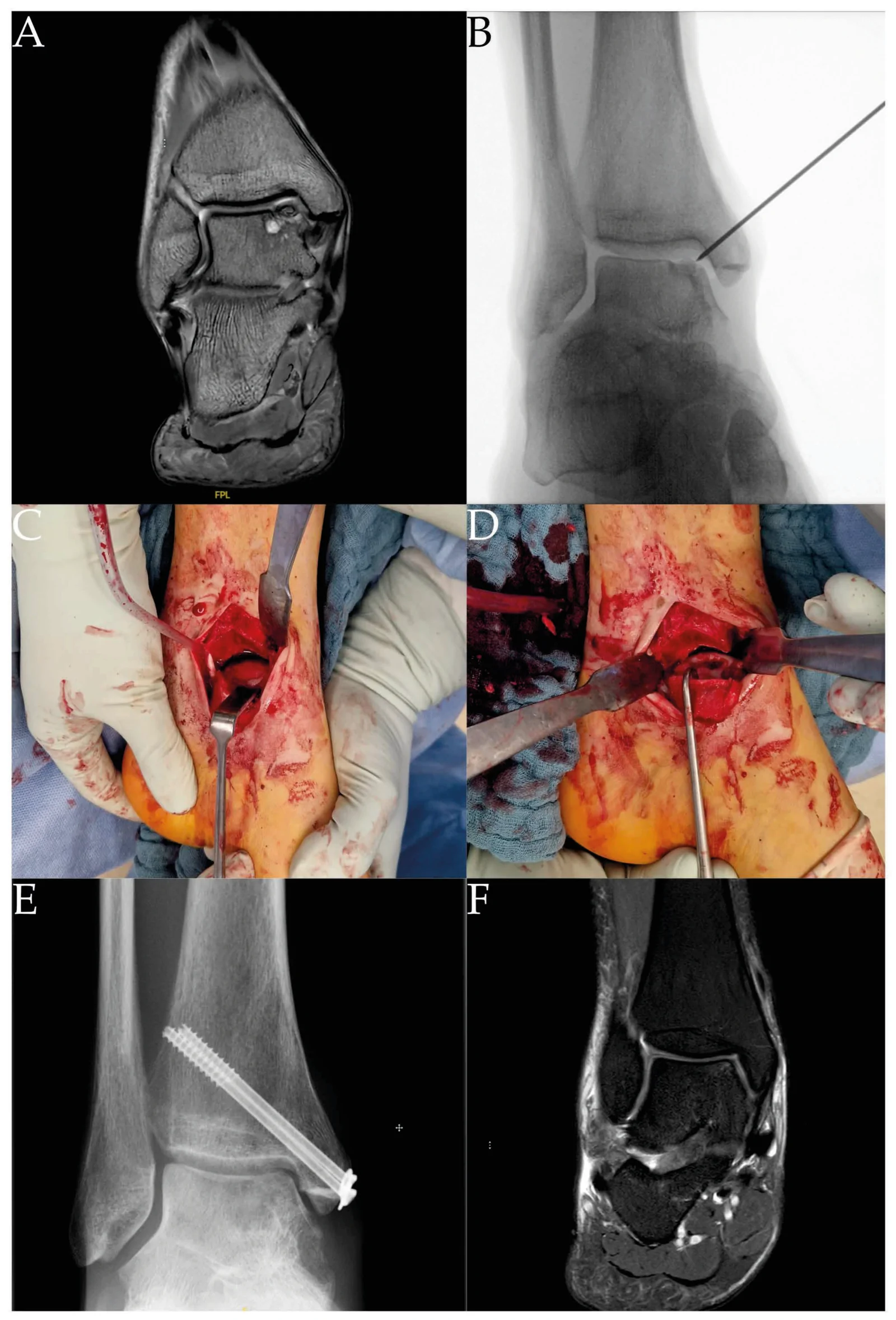
Exemplary case presentation, patient with stage 4 OD. (**A**): Preoperative MRI showing clearly visible dissection; (**B**): intraoperative X-ray imaging prior to osteotomy; (**C**): post-osteotomy and debridement, showing the defect area; (**D**): filling of the defect area with iliac crest cancellous bone and coverage with Epiflex, stabilization by spraying with fibrin; (**E**): postoperative X-ray; (**F**): MRI of the same patient 14 months postoperatively, still showing slight bone edema but good visualization of the cartilage-bone junction; the examination lies within the postoperative imaging window reported in Table 3.

**Table 1:** Comparison of the operative regenerative treatment options in OD.

Treatment Method	Description	Advantages	Disadvantages	Indications
**MACT**	Two-step procedure Extraction of chondrocytes and combination with a scaffold	- Promising long-term outcomes for cartilage regeneration - Potential for improved cartilage quality	- Extended rehabilitation period - Long healing process	- Suitable for young patients - Effective for large cartilage defects
**OATS**	Autotransplantation of chondrocytes	- Good graft integration with host tissue - Relatively quick recovery	- Limited by donor site availability - Potential mismatch in cartilage quality	- Effective for small-to-moderate defects - New defect at donor site
**Autocart and minced cartilage**	Autotransplantation of chondrocytes and processing with platelet-rich plasma or fibrin or thrombin	- Good biocompatibility - Good healing potential	- Limited by donor site availability - Potential mismatch in cartilage quality	- Suitable for smaller to medium defects - Preferred in younger patients
**AMIC^®^**	Microfracturing or Autotransplantation of cancellous bone	- Good biocompatibility - Good healing potential	- Porcine collagen membrane	- Effective for large cartilage defects - Long-term stable results

**Table 2:** FADI and FADI-Sport.

Foot and Ankle Disability Index Items	Foot and Ankle Disability Index Sport Items
Standing	Running
Walking on even ground	Jumping
Walking on even ground without shoes	Landing
Walking up hills	Squatting and stopping quickly
Walking down hills	Cutting, lateral movements
Going up stairs	Low-impact activities
Going down stairs	Ability to perform activity with your normal technique
Walking on uneven ground	Ability to participate in your desired sport as long as you would like
Stepping up and down curves	
Squatting	
Sleeping	
Coming up on your toes	
Walking initially	
Walking 5 min or less	
Walking approximately 10 min	
Walking 15 min or greater	
Home responsibilities	
Activities of daily living	
Personal care	
Light to moderate work (standing, walking)	
Heavy work (push/pulling, climbing, carrying)	
Recreational activities	
General level of pain	
Pain at rest	
Pain during your normal activity	
Pain first thing in the morning	

**Table 3:** Individual patient data. All scores are raw point values (FADI maximum 104 points, FADI Sport maximum 32 points). None of the patients had undergone previous surgery of the affected ankle or a previous cartilage repair procedure. Clinical follow-up was performed at a mean of 13 months (range 10–14 months) in every patient. FU = follow-up; n/a = not available.

Pt	Age/Sex/Side	Etiology	Volume (cm^3^)	FADI Pre → Post	FADI Sport Pre → Post	MRI (Months)	MOCART 2.0	Complications/Hardware Removal	Symptoms at FU
1	64/m/right	post-traumatic	3.01	41 → 104	14 → 32	11	70	not yet	none
2	27/m/left	idiopathic	2.98	33 → 103	5 → 32	12	65	12	none
3	62/m/right	post-traumatic	3.45	23 → 103	0 → 32	14	75	13	none
4	43/f/right	post-traumatic	5.02	28 → 68	3 → 9	14	50	13	persistent
5	57/f/right	post-traumatic	4.87	23 → 98	1 → 31	declined	n/a	declined	none
6	22/m/right	idiopathic	4.23	39 → 103	1 → 31	12	65	12	none
Mean	45.8/4 m 2 f/5 right	4 post-traumatic/2 idiopathic	3.93	31.2 → 96.5	4.0 → 27.8	12.6	65 ± 9.4	4/6 removed	5/6 free

**Table 4:** Baseline parameters of the analysis.

Age (Years)	Sex (Male/Female)	Operation Side (Right)	FADI Pre-Operation	FADI Sport Pre-Operation
45.8 (22–64)	66.7%/33.3%	83.3%	31.17 (+/−7.8)	4.0 (+/−5.22)

**Table 5:** Outcome parameters of the analysis.

FADI Post-Operation	FADI Sport Post-Operation	Freedom from Complaints	Procedure Time (min)	Revision of Cartilage Reconstruction	Number of Postoperative Consultations
96.5 (+/−14.12)	27.83 (+/−9.24)	83.3%	73.8 (+/−16.6)	0%	5.8 (+/−5.3)

**Table 6:** Distribution of the individual MOCART 2.0 Ankle Score subcategories for the five patients with available MRIs. Patient 5 is not listed because that patient declined a further MRI examination because of claustrophobia. The maximum attainable score for each subcategory is given in the last row.

Patient	Volume Fill	Integration	Surface	Signal Intensity	Bony Defect/Overgrowth	Bone Marrow Edema	Subchondral Cysts	Total
1	20	10	5	10	10	5	10	70
2	15	10	0	10	20	5	5	65
3	15	15	5	10	20	5	5	75
4	15	10	0	10	10	0	5	50
6	15	15	5	10	10	5	5	65
Mean	16.0	12.0	3.0	10.0	14.0	4.0	6.0	65.0
Maximum	20	20	5	15	20	10	10	100

## Data Availability

The data presented in this study are available on request from the corresponding author due to privacy reasons.

## Abbreviations

The following abbreviations are used in this manuscript:

ccm	cubic centimeters
ECM	extracellular matrix
FADI	Foot and Ankle Disability Index
hADM	human acellular dermal matrix
MACT	Matrix-associated chondrocyte transplantation
OATS	osteochondral autologous transplantation method
OD	Osteochondrosis Dissecans
